# When Adolescents Become Dads: A Clinical-Qualitative Study on the Experience of Male Adolescents Attending a Prenatal Care Service

**DOI:** 10.1007/s10995-026-04302-y

**Published:** 2026-07-20

**Authors:** Juliana Vasconcellos Freitas-Jesus, Fernanda Garanhani Surita, Débora Bicudo Faria-Shutzer, Rodrigo Almeida Bastos, Egberto Ribeiro Turato

**Affiliations:** 1 Department of Obstetrics and Gynecology, School of Medical Sciences, University of Campinas, R. Tessália Vieira de Camargo 126, Cidade Universitária, Campinas, São Paulo 13083-887 Brazil; 2 Laboratory of Clinical-Qualitative Research (LCQR), School of Medical Sciences, University of Campinas, R. Tessália Vieira de Camargo 126, Cidade Universitária, São Paulo 13083-887 Campinas, Brazil; 3 Present Address: Faculty of Psychology, School of Life Sciences, Pontifical Catholic University of Campinas, Av. John Boyd Dunlop, s/n , SP Campinas, Brazil; 4 Present Address: Department of Professional Guidance, School of Nursing, University of São Paulo, Av. Dr. Enéas de Carvalho Aguiar, 419, São Paulo SP, Brazil

**Keywords:** Pregnancy in adolescence, Adolescent father, Qualitative research, Prenatal care

## Abstract

**Objectives:**

Adolescent pregnancy has garnered public interest due to its implications for health, social dynamics, and economic outcomes. Fatherhood contributes to the formation of the identity of young men, particularly during their transition to adulthood. Prenatal care presents a valuable opportunity to involve adolescent fathers in parenting and supporting their pregnant partners. This study aims to investigate how adolescent fathers perceive fatherhood while attending a specialized prenatal clinic.

**Methods:**

This qualitative study focused on 14 expectant adolescent fathers aged up to 19 years, recruited from a specialized prenatal care clinic at a tertiary hospital in Brazil. Employing a purposive sampling strategy, we utilized in-depth interviews, sociodemographic questionnaires, and field diaries for data collection. Data analysis involved content analysis, contextualized within the framework of Health Psychology.

**Results:**

We raised four categories: (1) “Myself, a dad?!”: reactions to a positive pregnancy test and the young men’s concepts of fatherhood; (2) The imagined relationship with the baby and expectations for the future; (3) The new dynamics in the relationship with the female adolescent during pregnancy; and (4) The influence of the social circles on the development of fatherhood: the family of origin and the friends.

**Conclusions:**

The findings underscore the importance of integrating adolescent fathers into healthcare initiatives, addressing critical life-course topics such as family planning, bonding with the child, social support networks, and assistance for adolescent women. These insights highlight the potential for prenatal care settings to offer comprehensive support and guidance to young men navigating their parental roles.

## Significance

What Is Already Known on This Subject?

What This Study Adds?

Adolescent fatherhood can lead to negative socioeconomic consequences for young men, including lower education attainment, higher rates of early marriages, and premature entry into the workforce. In the context of best practices for prenatal care, actively engaging fathers in the pregnancy and childbirth process offers significant clinical and psychological benefits for the fathers themselves, as well as for the women and the infants. Consequently, it is essential to better understand the lived experience of expectant adolescent fathers attending prenatal care appointments.

The adolescent males interviewed acknowledged the need for behavioral, idealized their future relationships with their babies, and reported positive shifts in their relationships with their partners. They also highlighted the crucial role of family and friends in fostering a positive outlook on fatherhood. However, perceptions of fatherhood varied based of their circumstances. A clear contrast exists between those who planned the pregnancy, those who did not plan it but anticipated a lasting relationship with their partner, and those who did not plan it and doubted the longevity of the relationship.

## Introduction

Adolescent pregnancy has garnered significant public interest because of its associated risks to the health, social, and economic outcomes of young women(Ganchimeg et al., 2014; Hodgkinson et al., 2014). The World Health Organization (WHO) estimates that 16 million adolescents give birth annually, frequently in contexts where pregnancy may not have been planned or desired (World Health Organization, 2011b). In Brazil, women between 15 and 19 years old account for a substantial proportion of the overall fertility rate (17.4%). Among this population, 37.1% continue to reside with their families of origin, while 44.7% live with a partner or serve as the head of the household in the family (Instituto Brasileiro de Geografia e Estatística, 2017). Investigating the male partner has been identified as a crucial approach to understanding adolescent pregnancy, especially when the partner is also an adolescent (Hill et al., 2015; Lemay et al., 2010; Lohan et al., 2010; Paranjothy et al., 2009; World Health Organization, 2011b).

Adolescent fatherhood can entail severe socioeconomic consequences for young men, such as reduced educational attainment, increased rates of early marriage, and premature entry into the workforce (Assini-Meytin and Green 2015; Fletcher and Wolfe 2012a; Lee et al. 2013). In a systematic review regarding the antecedents and consequences of adolescent fatherhood, the authors indicated that teen fathers predominantly originate from minority and low-income households, exhibit lower academic achievement, participate in higher rates of delinquent activities (such as vandalism), and frequently associate with peer groups involved in antisocial behavior. (Bamishigbin et al., 2019). However, the experience of fatherhood varies across distinct contexts. For example, the adolescent might be in a stable relationship with his partner and actively assume responsibility within the family; conversely, the pregnancy may result from a casual encounter or occur under conditions of heightened vulnerability, such as involvement in crime (Shade et al., 2013).

The experience of becoming a father may contribute to a young man’s sense of identity, especially given that it occurs during a period of intense transformation into adulthood. The WHO defines adolescence as the period between 10 and 19 years of age—a developmental stage characterized by vulnerabilities that demand explicit and specific actions within healthcare programs and policies (Dick & Ferguson, 2015; Sawyer et al., 2018) However, in Brazil, male adolescents are rarely integrated into the healthcare system, even within primary care services. This lack of institutional engagement further exacerbates their vulnerability due to restricted access to healthcare, educational initiatives, and psychosocial support. Beyond these systemic challenges, when a young man accompanies his pregnant adolescent partner to healthcare services, it presents a pivotal opportunity for practitioners to offer targeted support and guidance (UNFPA, 2015; World Health Organization, 2011a).

In terms of best practices in prenatal care, actively involving fathers offers profound clinical and psychological benefits not only for the men themselves but also for the mothers and infants. Approaching fathers during prenatal education can significantly improve father-infant attachment, through both virtual and in-person interventions, as demonstrated by a clinical trial in Iran(Doaltabadi & Amiri-Farahani, 2021). Similarly, midwives in Australia perceive that their role encompasses supporting paternal well-being and family functioning, noting that the presence of fathers in maternity services makes women feel supported, provided there is no risk of domestic violence (Wynter et al., 2021) In Brazil, results from a qualitative study investigating paternal involvement in prenatal care and childbirth indicated that men’s participation is highly significant and warrants deeper exploration, particularly in light of evolving models of masculinity in contemporary society. In this sense, midwives and the broader healthcare team serve as essential catalysts for fostering greater paternal adherence to this process. (Lima et al., 2021)

Considering the multifaceted vulnerabilities these young men encounter, alongside the potential for healthcare interventions to foster active parenting and partner support during prenatal care, it is essential to better understand the lived experiences of expectant adolescent fathers attending prenatal care appointments. We aimed to explore the meanings that male adolescents attribute to their experience of fatherhood when attending a specialized prenatal clinic to accompany their pregnant adolescent partners, to accompany their pregnant partners, specifically examining their emotional experiences regarding the infant, their partners, their families, and themselves.

## Methods

This is an exploratory study applying the clinical-qualitative method, a research approach developed to collect and analyze qualitative interviews within healthcare contexts. (Faria-Schutzer et al., 2019; Turato, 2013) We conducted in-depth interviews, applied the clinical-qualitative content analysis, and followed the COREQ Checklist. (Tong et al., 2007) The study was approved by the local research ethics committee (protocol number: 57823816.4.0000.5404), in accordance with the Declaration of Helsinki and national regulations. All participants provided written informed consent prior to their inclusion in the study. To ensure confidentiality, all identifying details were omitted, and participants’ names were replaced with numerical identifiers.

### Participants and Setting

We interviewed male adolescents based on the following inclusion criteria: aged 19 years or younger; being the partner of a pregnant adolescent; voluntarily attending the outpatient clinic as a companion; and acknowledging paternity. The exclusion criteria were an unwillingness to have the interview audio-recorded and any health condition that precluded verbal communication during the interview. We utilized purposive sampling, with sample size determined by theoretical saturation (Glaser & Strauss, 1999).

The participants were recruited from an outpatient prenatal care clinic specialized in adolescents, located within a university tertiary hospital in Brazil. The hospital is a reference center for more than 100 municipalities and 5 million inhabitants, serving the public health sector exclusively. Outpatient care was provided twice a week by a multidisciplinary team (comprising obstetricians, nurses, a social worker, and a psychologist). Prenatal care in Brazil is typically delivered by physicians and obstetric nurses, whose roles correspond closely to those of midwives.

### Instruments

Data collection instruments included: (1) semi-directed interviews with open-ended, in-depth questions; (2) sociodemographic questionnaires; and (3) field diaries. For the interviews, we used the trigger question, “How are you experiencing this pregnancy?”. The interview guide covered domains such as the participant’s relationship with the pregnant adolescent, their family of origin, their feelings and expectations about the baby, and their friendships. Table 1 shows the complete interview guide. We collected sociodemographic data, including age, educational attainment, paid work, and household composition. Field diaries were utilized to record the researcher’s perceptions after each interview, including participants’ non-verbal communication.

**Table 1:** The interview script

Interview Script – Open-ended and in-deep questions for the interviews	
Trigger question: Tell me how you are experiencing this pregnancy. What do you think when the baby comes to your head? How is it for you to come here for prenatal consultations? How are you your relationship with the baby’s mother? And with your family? And with your friends? Considering your experience with pregnancy, how do you think your head and your life will look like?	

**Table 2:** The seven steps of the clinical-qualitative content analysis

Seven steps of the clinical-qualitative content analysis	Description
1. Editing materials	Complete transcription of the interviews from the audio recordings.
2. Floating Reading	Multiple readings of the transcribed material in order to absorb the contents of the interviews, but not yet starting the categorization process.
3. Comments and Impressions on the Interviews Set	Annotating the transcribed material with initial reflections on the whole set.
4. Emergence of the Meaning Cores	Initial agglutination of the psychological meanings that emerge from the set of interviews and which are fundamental to the goals of the research.
5. Categorization	Definition of the categories through the articulation of the different meaning cores, containing excerpts of the interviews that represent the ideas explored in each of the categories.
6. Discussion	Discussion of the categories using the scientific literature and the theoretical framework chosen by the researcher to interpret the results.
7. Validation of the categories by the peers	Presentation to and discussion of the categories with the research partners, specialists on the method, to validate the results.

### Recruiting and Data Collection Procedures

We collected data weekly between September 2016 and August 2017. The first author (JVFJ), a psychologist with training and experience in adolescent research and clinical care, performed recruitment and data collection. She underwent a six-month immersion (acculturation process) in the outpatient prenatal clinic preceding data collection to familiarize herself with its routines and institutional microculture (e.g., healthcare team and patient profiles, and operational workflows). Two pilot interviews were conducted before formal data collection to refine the interview guide, and these were excluded from the final sample.

We performed recruitment in the waiting room when the adolescent fathers attended prenatal appointments with their adolescent partners. Interviews were conducted in a private room, where the researcher explained the study’s objectives, design, and ethical terms before obtaining written consent. The interviews were audio-recorded and subsequently transcribed verbatim.

In total, we recruited 18 adolescents; 14 were interviewed, and four refused to participate, stating that they did not feel comfortable discussing fatherhood. The interviewer emphasized her interest in the adolescents’ genuine experiences and stressed that there were no correct or incorrect answers, ensuring a non-judgmental environment. After completing the interview guide, the interviewer asked the participants if they wished to add any further reflections.

### Data Treatment and Analysis

The interviews were transcribed verbatim. For the treatment and analysis of the data, we followed the seven steps of Clinical Qualitative Content Analysis(Faria-Schutzer et al., 2019) This analysis method includes: (1) editing the material; (2) free-floating reading; (3) constructing units of analysis; (4) identifying cores of meaning; (5) consolidating categories; (6) discussing the topics; and (7) validation, as described in Table 2. Peer validation of the categories (steps 6 and 7) was performed with coauthors and researchers from the Clinical-Qualitative Research Laboratory (LPCQ) and the Research Group on Reproductive Health and Healthy Habits (SARHAS), both based at the School of Medical Sciences of the University of Campinas.

### Trustworthiness

We employed four strategies to ensure the trustworthiness of the research: credibility, transferability, dependability, and confirmability. (Shenton, 2004).

The credibility of the results was enhanced by establishing early familiarity with the research setting during the six-month immersion (acculturation process) and through prolonged engagement with the data. We emphasize the importance of establishing rapport with participants by using accessible language, ensuring privacy, and employing interactive questioning (rephrasing questions to elicit richer detailed descriptions from the adolescents). The categories that emerged from the data analysis were validated by the research team to avoid bias. To ensure transferability, we used purposive sampling by inviting consecutive eligible adolescent fathers attending the outpatient prenatal clinic. Dependability and confirmability were strengthened through external validation and peer debriefing involving four authors specialized in the clinical-qualitative method and one author with extensive experience heading the outpatient prenatal care clinic where the study was conducted. Regarding transferability, we recognize that our findings represent one possible interpretation among others; however, this does not diminish the validity of the data, as our results provide valuable insights transferable to similar clinical and sociocultural contexts.

## Results

The participants spontaneously attended prenatal care and were interviewed during the pregnancy of their first child. The 14 interviews lasted between 30 and 90 min. The participants’ ages ranged from 16 to 19 years. Three participants reported that they had planned the pregnancy, and eight were currently cohabiting with their pregnant adolescent partners. Furthermore, eleven participants had been in a relationship with their partners for more than one year. Table 3 presents the complete sociodemographic characteristics of the participants. Although all adolescents expressed a strong interest in maintaining a relationship with their partner and the infant in the future, they possessed only vague ideas about their daily responsibilities once the child was born.

**Table 3:** Sociodemographic data of the adolescent fathers (*n* = 14), Campinas, Brazil, 2017

		Age^1^	Schooling	Household composition	Occupation	Pregnant Partners		
						Age^1^	GA^2^	Relationship duration
Unplanned Pregnancy	P1	16	High school	Father, mother, brother (13y), sister (5y)	Unemployed	16	28	1y6m
	P2	19	High school	Pregnant partner	Formal work	17	35	1y1m
	P3	18	High school	Mother	Formal work	16	24	1y7m
	P4	18	High school	Pregnant partner, mother, grandmother, sister (16y), brother (11y)	Formal work	15	18	3y
	P5	19	High school	Pregnant partner	Informal work	19	22	4y
	P6	19	High school	Mother, 2 brothers (18y and 13y)	Informal work/criminality	15	18	2y
	P7	19	High school	Grandmother (55y)	Informal work	14	28	1y2m
	P8	18	High school	Pregnant partner	Informal work	16	4	6 m
	P9	17	Elementary	Father, mother, brother (14y)	Formal work	15	20	1y2m
	P10	18	High school	Pregnant partner, father, mother, uncle, sister (12y)	Criminality	14	12	6 m
	P11	17	Elementary	Pregnant partner	Informal work	14	28	7 m
Planned Pregnancy	P12	19	High school	Pregnant partner	Formal work	17	33	5y
	P13	18	High school	Mother and 8 brothers/sisters	Unemployed	16	32	4y
	P14	18	Elementary	Pregnant partner, in-law relatives (father, mother, 2 brothers, niece (3y))	Informal work	15	35	1y

^1^Years old; ^2^GA – Gestational Age (weeks); *P* participant; *y* years; *m* months

The majority of the interviewed adolescents lived in urban areas and belonged to low-income family socioeconomic backgrounds. In the Brazilian context, this reality can carry a series of implications regarding labor market entry, educational prospects, professional qualification, household composition, gender relations, and the young men’s perceptions of experiencing fatherhood at an early age, all of which are described below.

Regarding entry into the labor market, nine participants were unemployed or engaged in informal work, with two declaring involvements in criminal activities such as drug trafficking and theft. Some respondents reported working since childhood, including Participant 12, who began working in a local market at age nine before entering the gardening sector. Furthermore, the involvement of two participants in criminal activity reflects the severe structural vulnerabilities, economic constraints, and adverse social conditions that shape the life trajectories of these young men within the Brazilian context.

Regarding educational perspectives, 11 participants had already completed high school close to the expected age of graduation. Some expressed a desire to pursue higher qualifications, such as technical courses or higher education, though they voiced concerns about the feasibility of initiating these plans due to the pregnancy.

In terms of household composition, eight adolescents were living with their pregnant partner, three of whom resided in homes shared with other family members, such as parents or siblings. Six participants lived within their families of origin, which comprised various structures, such as single-parent households or grandparents acting as the primary caregivers for the young man.

Four thematic categories emerged from the data: (1) “Myself, a dad?!”: reactions to a positive pregnancy test and the young men’s concepts about fatherhoods; (2) The imagined relationship with the baby and the expectations about the future; (3) The new dynamics in their relationship with the female adolescent in the face of pregnancy; and (4) The influence of the social circles in constituting fatherhood: the family of origin and the friends.

### “Myself, a Dad?!”: Reactions to a Positive Pregnancy Test and the Young Men’s Concepts of Fatherhood

This category presents the participants’ reactions upon learning of the pregnancy and their concepts regarding the meaning of fatherhood, including their accounts of the behavioral changes required to assume a paternal role. The findings reveal idealizations of adulthood, feelings of unpreparedness, and a perceived incompatibility between wanting to remain a teenager and needing to assume parental responsibilities; conversely, some adolescents had already undertaken responsible roles within their families of origin.

When faced with an unplanned pregnancy (*n* = 11), the adolescents reported feelings of shock and worry. During the interviews, they referred to themselves as “young dads,” demonstrating that they felt unprepared for pregnancy and fatherhood. This reported sense of unpreparedness prompted them to make significant personal changes to better enact the fatherhood they idealized during the prenatal period. For example, many of the activities they engaged in before the pregnancy—such as drug and alcohol consumption, involvement in crime, and going to parties—were viewed as incompatible with their new role as a father. The young fathers reported these personal changes as positive—characterizing them as a period of maturation—but also as a source of regret involving a series of abdications. They also manifested a pressing need to search for a first job or secure better employment.

*“Ah*,* a young dad… At first*,* I was a bit scared. Because*,* oh my… Myself? Becoming a dad?! Now I have to keep going*,* right? […] I felt upset with myself*,* because now was not the right time*,* I had many things planned* [silence].*”* (Participant 1).

*“Myself arrested?! Leaving my girlfriend with the baby*,* and myself arrested. That is why I stopped involving myself with crime… The child without a dad… The dad died after robbing many times* [silence].*”* (Participant 6).

In navigating this feeling of being unprepared upon discovering the pregnancy, the adolescents started a process of psychologically elaborating their role, attempting to identify their duties and identity as a father. They described three main roles they attributed to themselves: (1) financial provision for both the pregnant adolescent and the infant; (2) transmission of personal values to their children; and (3) maintenance of a close relationship with the child. They talked about being good role models for their children, noting that changing their behavior wa**s** indispensable. The adolescents who did not intend to maintain the relationship with their partner likewise discussed behavioral modification; however, they expressed these changes as something painful.

*“I drank a lot every day. After she got pregnant*,* I got more focused on my home. I was able to save some money and I stopped being so impulsive! So*,* this pregnancy knocked some sense into me. It really taught me to have a different attitude. […]. And I will pass on to my child all the knowledge I got*,* that I could learn so far”* (Participant 2).

The adolescents who planned the pregnancy recalled the discovery with great joy. In these instances, family planning and professional stabilization occurred before the pregnancy and coincided with a trajectory where the young man was already assuming greater responsibilities within his family of origin. These adolescents were already part of the workforce and maintained a stable, long-term relationship with their partner. Consequently, they did not view behavioral changes as imperative, but rather anticipated that their daily routine would remain similar to their current lifestyle. Therefore, the arrival of a baby was not perceived as worrisome, but rather as a source of great joy.

*“The pregnancy did not happen without me or her wanting*,* something mandatory so that we might stay together. Both of us wanted*,* right? The two of us were going to stay together.”* (Participant 12).

### The Imagined Relationship with the Baby and Expectations for the Future

The adolescents manifested a bond with the baby beginning in pregnancy. These fathers-to-be thought about the infant and imagined what daily life would be like after the birth. The accounts were permeated predominantl**y** by affection and joy, and the young men talked about involving themselves in the daily care of their babies. However, their conceptions of the routine with the baby were still vague, and the findings also indicated that some youths had difficulty imagining the baby after birth. Some adolescents also discussed anxiety and the challenge of conceptualizing their future relationship with the child.

*“Ah*,* to bathe*,* change diapers*,* feed with the bottle*,* taking care*,* giving everything the child needs. The child will cry during the night*,* and sometimes my partner will be tired*,* and I can help her.”* (Participant 7).

*“Ah*,* this time has been very exciting because it is the first baby. And it has also been … scary! I don’t know how to look after the baby”* (Participant 4).

When reflecting on the relationship with their future children, the young fathers evoked experiences with their own fathers to shape their own approach to fatherhood. The adolescents who reported deprivation, neglect, a sense of abandonment, or the absence of **a** father figure expressed a strong desire to offer their own children a different experience. Conversely, those who had positive male role models sought to replicate those behaviors.

*“My stepfather is the father figure I have. He provides for the family*,* gave up his own friends (…) and stayed with my mom (…). He also began to act with more responsibility.”* (Participant 2).

Regarding their own future, fatherhood served as a motivation to pursue dreams that seemed distant before. Conversely, some adolescents noted that fatherhood had delayed, impaired, or altered their life plans. The plans they make for themselves now are guided by the necessity of becoming good role models for their children. Some adolescents envisioned continuing their studies, while others had already discontinued their studies due to a lack of motivation.

*“Because of the pregnancy*,* I will always want to become a better person*,* because before I was in my comfort zone*,* like*,* here I am*,* everything is good. Now I think about improving. Because it will be important for us.”* (Participant 5).

### The New Dynamics in the Relationship with the Female Adolescent During Pregnancy

Some adolescents reported that their relationship with their pregnant adolescent underwent important changes after learning of the pregnancy; specifically, fights decreased in intensity and frequency, the relationship becam**e** more stable, and they felt motivated to offer support to their partners. The desire to maintain the relationship with the partner can evoke feelings of hope and motivation to build a family and devise a life plan, despite underlying ambivalence and concern about playing this new role.

*“We are not doing that thing of fighting*,* splitting*,* and that’s it. Now*,* we must think that we will have a daughter. We must talk*,* it is not worth fighting for random stuff. And our relationship also matured a lot.”* (Participant 5).

*“We need each other now*,* right? Because if there is no support from the partner*,* who will support her?* [Silence] *Obviously*,* the family is supporting*,* but I am the one who has to offer support.”* (Participant 1).

On the other hand, some of the interviewees felt impelled to maintain their relationship with the pregnant adolescent, either driven by their own sense of obligation or by pressure from their parents. In these cases, they felt anxious and unprepared to play that role, particularly when they doubted the longevity of the relationship.

*“Her mother said that she would find a way to marry us… A marriage is no good*,* fights*,* betrayals. One kills the wife. It is a horrible thing.”* (Participant 11).

### The Influence of the Social Circles in the Constitution of Fatherhood: The Family of Origin and the Friends

When discussing the moment of telling their parents about the pregnancy, the most common feelings reported by the participants were fear and anxiety. Their parents’ reactions varied according to the family context. Some participants reported that their parents were surprised, shocked, and disappointed, while others reported happiness and joy regarding the arrival of a grandchild. When the relationship with the parents was distant, the participants reported indifference. Nonetheless, the adolescents manifested a desire to be recognized by their relatives as responsible fathers and felt supported by their parents in assuming this mature new role.

*“Ah*,* her mother talked about taking it out* [referring to a clandestine abortion] *‘You must take it off!’*,* she said*,* and started to cry. Her father was very angry.”* (Participant 3).

*“Will I be able to be a good father for my son? … Will I be able to be an excellent husband for her? I spoke to my mother*,* and she said that I should not think like that*,* that I would make it.”* (Participant 7).

*“To be honest*,* I was never really in touch with my family*,* neither my father nor my mother. But now they got closer to me […]. My mother never kept in touch*,* and never bothered much either.”* (Participant 5).

Regarding their friendships, the adolescents noted that some of their friends distanced themselves when they found out about the pregnancy. While this was sometimes viewed as advantageous, as it allowed them to identify their true friends among those who remained close, it was also a source of discontentment, making it appear as though fatherhood constituted a barrier to maintaining those ties. Conversely, for fathers whose friends also desired to build a family, fatherhood served as a source of greater shared happiness. Finally, some participants described negative examples of fatherhood among their peers that they wished to avoid, specifically citing instances where friends did not assume fatherhood or maintained a distant relationship with their children.

*“I still have some friends. Some of them disappear*,* right? I know who is a real friend*,* you know? Those who stayed.”* (Participant 3).

*“This friend of mine*,* who got in a fight with his woman […]*,* this was something that affected me a lot*,* I could not process that. His wife got pregnant*,* had the child … and he simply gave it all up! […] I get really upset with that!”* (Participant 2).

*“They* [two close friends who are together] *will also marry soon*,* and they already have plans for a daughter*,* so we are all in the same place and with the same mind. We are all in the same track* [laughter]’ (Participant 5).

## Discussion

This study investigated the experiences of expectant adolescent fathers who voluntarily accompanied their partners to a prenatal care service specializing in adolescent pregnancies. This sample is highly relevant because it reflects real-world clinical practices in prenatal care, thereby broadening the understanding of maternal-child healthcare to include young fathers as essential components of the family unit who play a crucial role in supporting both the woman and the infant. In summary, the adolescents highlighted the necessity of behavioral modifications (e.g., reducing substance abuse, ceasing involvement in criminal activities, and limiting attendance at parties or social outings with friends). They likewise idealized their future relationships with the baby, reported positive changes in their relationships with their partners, and indicated the importance of family and friends in fostering a positive outlook on fatherhood. However, a stark contrast emerged between the perceptions of fatherhood among those who planned the pregnancy, those who did not plan it but anticipated a lasting relationship with the pregnant adolescent, and those who did not plan it and expressed doubts regarding the longevity of the relationship.

Among the 14 interviewed adolescents, 11 reported an unplanned pregnancy, highlighting gaps in male involvement regarding family planning. In Brazil, although 71% of adolescents report using condoms during their last sexual encounter, only 3.6% utilize double protection, suggesting that young men prioritize STI prevention over active pregnancy prevention, a role historically delegated to women (Alves et al., 2017; Borges et al., 2021). While international guidelines recommend providing contraceptive counseling to all adolescents regardless of gender (Ott et al., 2025), further research is needed to explore how adolescent fathers perceive their role in family planning, thereby broadening our understanding of gender dynamics in youth pregnancy.

The results indicate that some adolescents face challenges during the transition to fatherhood, as they may lack the psychological and social resources required to develop their identity as a father. Identity formation during youth is a process through which adolescents form a self-concept shaped by their psychological and cultural contexts. (McLean & Syed, 2015). The United Nations Children’s Fund (UNICEF) highlights the complexity of defining adolescence, noting that it contingent upon various biological and cultural factors. Furthermore, certain life events are traditionally associated with adulthood, including marriage and childbearing. (United Nations Children’s Fund (UNICEF), 2011) In this sense, parenting during adolescence serves as a catalyst that prompts adolescents to reflect on their identities more deeply. This creates an overlap between their transition from adolescence to adulthood and their transition into fatherhood, which can ultimately carry significant implications for the mental health of these young people.

Early marriage is a significant factor associated with adverse socioeconomic outcomes among adolescent fathers (Fletcher and Wolfe 2012b). This is particularly relevant in Brazil, where the 2024 Census recorded over 11,000 adolescent marriages; the state of São Paulo, the site of this study, led the country with 3,491 male and 9,531 female adolescent marriages (Instituto Brasileiro de Geografia e Estatística, 2024).Within our sample of 14 participants, eight were already cohabiting — three in multigenerational households and five independently — while six intended to do so. These life trajectories reveal an early assumption of family responsibilities, suggesting that their adolescence was shaped by cumulative systemic vulnerabilities, including low-income contexts, premature labor market entry, and restricted access to healthcare and family planning services.

While most participants expressed positive motivation to assume financial obligations, associating this transition with developing adult responsibility, they also reported severe insecurity regarding these demands. This reality underscores the urgent need to expand the debate on early marriage beyond preventive measures, implementing clinical and social interventions once pregnancy and cohabitation are established. Future research and public policies must prioritize comprehensive support tailored to these youths’ socioeconomic realities, focusing on educational and career continuity, parenting support, access to reproductive rights, and targeted discussions on gender roles to mitigate the risk of violence against women.

Furthermore, the perspective of the young men’s own parents emerged as a key factor in their reflections on becoming fathers. A cross-sectional study conducted with adolescent fathers in the United States demonstrated an association between higher paternal satisfaction and fewer depressive symptoms. Specifically, the study showed that adolescents with a higher degree of satisfaction in their family relationships and fewer conflicts with their parents were less likely to develop symptoms of depression (Hunt et al., 2015). Consequently, it is crucial to design family health planning interventions that include the relatives of these fathers-to-be, enabling them to serve as sources of support and act as facilitators during this transitional period.

The role of peer groups is also crucial, especially in the absence of family support. The experiences of friends serve as relatable benchmarks for young fathers, fostering a high degree of identification based on shared age and circumstances. (Prinstein, 2013) Consequently, identifying with peers may constitute a foundational element in the construction of paternal identity during youth.

Our results suggest that adolescent fathers previously involved in criminal activity may feel motivated to distance themselves from crime upon learning of the pregnancy. While some studies indicate that young fathers exhibit higher rates of delinquency or aggressive behavior compared to their childless peers (Assini-Meytin & Green, 2015; Hunt et al., 2015), there is no consensus on whether fatherhood serves as a turning point for criminal desistance (Berger & Langton, 2011; Edin et al., 2009), or act as a facilitator for subsequent offending (Na, 2016).

The discussion on youth criminality in Brazil must transcend individual choices to address complex systemic factors, such as structural racism, social inequality, precariously funded schools, and the economic allure of drug trafficking, which offer a perverse pathway to identity and financial insertion (Minayo & Constantino, 2012; Zalua, 2012). Nevertheless, our findings highlight a desire among these young men to abandon criminal paths upon entering fatherhood. To determine whether these intentions translate into sustained behavioral change, prospective studies are necessary to identify effective support strategies, leveraging the pregnancy period as a strategic entry point for healthcare services to provide guidance and structural support.

A primary limitation of this study is its focus on a specific developmental phase: adolescent fatherhood during pregnancy. The results reflect the expectations of these young fathers regarding their relationships with an unborn child, meaning that their perceptions may evolve following the birth and across the infant’s subsequent developmental stages. Additionally, we note that data collection occurred in 2017; while this historical timeframe may introduce certain temporal limitations, it does not invalidate the relevance of the findings. Finally, although our study’s reliance on a specific sociocultural and temporal context might restrict the generalizability of the data, these constraints do not diminish its capacity to offer valuable insights and to provide opportunities for reflection among healthcare professionals who serve this population.

During data collection, we encountered distinct challenges that warrant consideration in future research. Because this study focused exclusively on adolescent fathers who voluntarily attended a prenatal care service, the pool of eligible participants was inherently limited. Although accessing this specialized clinic did not legally require the presence of a guardian, many pregnant adolescents were accompanied instead by their mothers or other adult relatives. Ultimately, 18 adolescent fathers were approached, four of whom declined to participate without providing a reason. Among those interviewed, several initially exhibited a guarded or distrustful demeanor toward the researcher, necessitating the deliberate establishment of rapport prior to the interview. For instance, room furniture was rearranged to remove the physical barrier of the obstetrician’s desk between the interviewer and the participant, and conversational adjustments were made to employ more informal, accessible language.

## Final Considerations

Our participants pointed to several behavioral aspects that they needed to change in themselves to facilitate a better transition to fatherhood. These self-imposed changes may serve as motivations to become better fathers and adults; however, we noted that such strict and demanding expectations might prove unsustainable over the long term. In this sense, healthcare interventions may help them balance their internal expectations with sustainable personal goals.

The results of this study may encourage healthcare professionals to actively include and provide follow-up care for this population during prenatal care. The planning of these healthcare interventions should be directed towards encouraging active paternal engagement, supporting the pregnant adolescent, providing sexual education, facilitating life and family planning, and incentivizing young men to pursue further education and career development. New studies could investigate specific healthcare actions that support the transition into fatherhood among these young men, deepen the empirical understanding of peer dynamics and the role of social circles in shaping the concept of fatherhood, and explore methods to integrate their broader support networks to secure optimal social and health outcomes. Finally, future research should explore how adolescent fathers perceive their role in family planning to deepen the understanding of how traditional gender roles interact with adolescent pregnancy, particularly when it is unplanned.

## Data Availability

Data is available on request sent to the corresponding author.
